# The pathogenic biomarker alcohol dehydrogenase protein is involved in *Bacillus cereus* virulence and survival against host innate defence

**DOI:** 10.1371/journal.pone.0259386

**Published:** 2022-01-04

**Authors:** Devon W. Kavanaugh, Constance Porrini, Rozenn Dervyn, Nalini Ramarao

**Affiliations:** Micalis Institute, INRAE, AgroParisTech, Université Paris-Saclay, Jouy-en-Josas, France; Sam Higginbottom University of Agriculture, Technology and Sciences, INDIA

## Abstract

*Bacillus cereus* is a spore forming bacteria recognized among the leading agents responsible for foodborne outbreaks in Europe. *B*. *cereus* is also gaining notoriety as an opportunistic human pathogen inducing local and systemic infections. The real incidence of such infection is likely underestimated and information on genetic and phenotypic characteristics of the incriminated strains is generally scarce. We have recently analyzed a large strain collection of varying pathogenic potential. Screening for biomarkers to differentiate among clinical and non-clinical strains, a gene encoding an alcohol dehydrogenase-like protein was identified among the leading candidates. This family of proteins has been demonstrated to be involved in the virulence of several bacterial species. The relevant gene was knocked out to elucidate its function with regards to resistance to host innate immune response, both *in vitro* and *in vivo*. Our results demonstrate that the *adhB* gene plays a significant role in resistance to nitric oxide and oxidative stress *in vitro*, as well as its pathogenic ability with regards to *in vivo* toxicity. These properties may explain the pathogenic potential of strains carrying this newly identified virulence factor.

## Introduction

*Bacillus cereus* is an ubiquitous spore forming human pathogen. It is present in soil, foods, almost all surfaces in hospital settings, and human skin. It is the second leading cause of collective foodborne outbreaks in France after *Staphylococcus aureus* and the third in Europe [1–3]. *B cereus* was associated with 155 outbreaks, 1,636 illnesses and 44 hospitalizations in Europe in 2019 according to reports by 27-member states. *B*. *cereus* can induce two types of gastrointestinal diseases, leading to emetic or diarrhoeal syndromes. *B*. *cereus* can also cause severe systemic infections, especially in immunocompromised patients leading to patient death in approximately 10% of cases [4–9]. However, some *B*. *cereus* strains can cause severe and even fatal infections in healthy people [10]. The pathogenic potential of *B*. *cereus* is thus extremely variable, with some strains being harmless and others lethal [11].

*B*. *cereus* produces toxins such as Hbl, Nhe, and CytK that induce cell toxicity [12–14]. In addition, other factors such as HlyII, InhA1, CwpFM or Mfd have been implicated in *B*. *cereus* resistance against the host immune system [15–21]. These toxins provide an indication of the strain toxicity potential [13, 22–24]. However, these factors do not allow the discrimination of strains according to their pathogenicity. Indeed, several studies have shown that the Nhe production by hazardous strains is variable and that non-pathogenic strains can also produce it in large quantities [1, 24]. Moreover, these toxins do not appear to be suitable markers for strains causing non-gastrointestinal infections [22].

*B*. *cereus* strains that induced severe gastrointestinal or non-gastrointestinal disorders do not carry neither *hbl*, *ces*, *hlyII*, *cytK1* nor *cytK2* genes and did not produce the Nhe protein, implying that other still unknown factors were responsible for their pathogenicity [1, 11].

Accordingly, we have recently analyzed a large strain collection comparing strains that induced an infection (intestinal or otherwise) with non-pathogenic strains [11, 25]. The large strain screening allowed to identify a combination of four as yet undescribed biomarkers, wherein their presence/absence allows an accurate identification of clinical *B*. *cereus* strains [26]. Three of these genes are located on the bacterial chromosome, and the fourth one is located on a large plasmid in a region that could be defined as a novel pathogenicity island for *B*. *cereus* [27]. These findings constitute a huge step in the understanding of the *B*. *cereus* pathogenic potential and complexity and may provide tools to better assess the risks associated with *B*. *cereus* contamination. Among these genes, *adhB*, was identified as a leading candidate [26]. This *adhB* gene encodes an alcohol dehydrogenase-like protein (ADH). This family of enzymes is involved in oxidation-reduction biological process. ADH are involved in metabolic and physiological processes in a variety of organisms, including fermentative metabolism [28], the oxidation of alcohols as carbon and energy sources [29], protection against anaerobic stress [30], and maintenance of the intracellular redox balance [31].

In this study, the *adhB* gene was knocked out to better elucidate its function during *B*. *cereus* virulence. Our results demonstrate that *adhB* plays a significant role in resistance to nitric oxide (NO) and oxidative stress *in vitro*, as well as its pathogenic ability with regards to *in vivo* infection and toxicity. These properties may explain the pathogenic potential of strains carrying this newly identified virulence factor.

## Materials and methods

### Bacterial strains

This study includes 35 *B*. *cereus* strains isolated from human patients following systemic or local infections and 21 non-pathogenic strains (Table 1). The 35 strains of the clinical collection were isolated from patient samples (biopsy, blood culture, etc) from nine French voluntary hospitals between 2008 and 2014. The samples and information were collected for a previous study and were treated anonymously and thus not subjected to personal consent [22]. The non-pathogenic strains have been isolated from food, where no infection was reported in humans. They were further tested in cell and animal models and did not induce any pathologies [23, 25]. We have previously shown a correlation between cytotoxicity and virulence [11]. Nevertheless, although these strains had previously been shown to be weakly cytotoxic to human cells and to have reduced virulence in an insect infection model, this does not rule out their potential ability to produce symptoms in specific vulnerable populations (i.e. the elderly, immunocompromised, or premature/new-born babies).

**Table 1:** Characteristics of non-pathogenic (A) and clinical (B) strains.

A					
**Non-pathogenic strains**		**Source**	***adhB***		
INRA-PF_**S09**		Milk protein	0		
I13**_S10**		Cooked rice	1		
INRA-5_**S11**		Pasteurized zucchini puree	0		
INRA-C64_**S12**		Pasteurized vegetables	0		
ADRIA-I3_**S13**		Cooked foods	0		
INRA-BN_**S36**		Vegetable	1		
INRA-PA**_S37**		Milk protein	0		
INRA-A3_**S38**		Starch	1		
I23**_S39**		Cooked apple	0		
SB**_S40**		Soil from a vegetable field	0		
I11**_S41**		Cooked food	1		
INRA-C1_**S42**		Pasteurized vegetables	0		
INRA-C46_**S43**		Pasteurized vegetables	0		
INRA-SL_**S44**		Soil	0		
INRA-SO_**S45**		Soil	0		
INRA-BC**_S47**		Vegetable	1		
I2_**S48**		Dried fruit	0		
INRA-BL_**S49**		Vegetable	0		
ADRIA I21_**S50**		Cooked foods	0		
INRA-SV_**S51**		Soil	0		
WSBC 10204_**S52**		Pasteurized milk	0		
B					
**Clinical strains**	**Age of patients**	**Type of sampling**	**Symptoms**	**Outcomes**	***adhB***
09CEB13BAC**_S6**	Premature newborn	Blood culture	Brain abscess	Recovery	1
09CEB14BAC_**S7**	Premature newborn	Blood culture	Bacteremia	Recovery	1
09CEB33BAC_**S8**	Newborn	Axilla-later feces	Skin infection	Recovery	1
12CEB31BAC_**S14**	Premature newborn	Blood culture	Organ failure and pulmonary and cerebral abscesses	Death	1
13CEB06BAC_**S15**	86	Blood culture from catheter	Heart failure, ventilator-associated pneumonia, ischemic stroke	Recovery	1
09CEB11BAC_**S16**	Premature newborn	Blood culture	Meningitis, infection in the liver, both lungs	Death	1
09CEB16BAC_**S17**	Newborn	Umbilical	Local colonization	Recovery	1
12CEB30BAC_**S18**	Premature newborn	Blood culture	Sepsis	Recovery	1
12CEB40BAC_**S20**	63	Blood culture	Bacteremia and central venous catheter-linked infection	Recovery	1
12CEB46BAC _**S21**	61	Blood culture	Sepsis (patient with an acute myeloid leukemia)	Recovery	1
12CEB47BAC_**S22**	43	Blood culture	Bacteremia	Recovery	1
12CEB51BAC_**S23**	60	blood culture	Sternum abscess, absent fever	Sequela of osteitis	1
13CEB01BAC_**S24**	31	Prosthesis from tibia	No clinical sign of infection	Recovery	1
09CEB12BAC_**S53**	Premature newborn	Cerebrospinal fluid	Meningitis, infection in the liver, both lungs	Death	1
09CEB34BAC_**S59**	Premature-newborn	Stomach-tube feeding	Premature birth	Recovery	1
09CEB36BAC_**S61**	Premature-newborn	Central venous catheter	Bacteremia	Recovery	1
12CEB34BAC_**S64**	80	Thoracentesis	Pulmonary infection	not known	1
12CEB37BAC_**S90**	30	Blood culture	Endocarditis	Death	1
12CEB38BAC_**S91**	65	Blood culture	Sepsis	Death	1
12CEB39BAC**_S92**	54	Blood culture	Sepsis	Recovery	1
12CEB42BAC_**S94**	63	Blood culture	Bacteremia and central venous catheter-linked infection	Recovery	1
12CEB43BAC_**S95**	63	Blood culture	Bacteremia and central venous catheter-linked infection	Recovery	1
12CEB44BAC_**S96**	34	Blood culture	Bacteremia	Recovery	1
12CEB45BAC_**S97**	newborn	Blood culture	Kidneys and urinary infections	Recovery	1
12CEB48BAC_**S98**	66	Blood culture	Bacteremia (patient with a colorectal cancer)	Recovery	1
12CEB49BAC_**S99**	24	Blood culture+ skin infection	Sepsis and aplastic anemia caused by drugs	Recovery	1
12CEB50BAC_**S100**	77	Blood culture	Bacteremia (patient with breast cancer)	Recovery	1
12CEB52BAC_**S101**	40	Blood culture	Bacteremia (immunocompromised patient)	Recovery	0
13CEB03BAC_**S102**	76	Blood culture	Community acquired pneumonia	Recovery	1
13CEB07BAC_**S105**	24	Blood culture	Abdominal pain, shivering, vomiting, fever, diarrhea	Recovery	1
13CEB09BAC_**S106**	85	Liver abscess	Sepsis, hepatitis c and liver abscess, abdominal pain, diarrhea	Recovery	1
13CEB30BAC_**S107**	not known	Blood culture	Nausea, abdominal pain and vomiting	not known	1
14CEB16BAC_**S114**	Premature newborn	Blood culture from peripheral veins	Septic shock, multiple organ failure, pulmonary and cerebral abscesses	Death	1
14CEB17BAC_**S115**	Premature newborn	Bronchial aspiration (lung)	Septic shock and pneumonia	Death	1
			pulmonary necrotic abscesses, recurrent pneumothorax		
14SBCL987_**S116**	not known	Biopsy (kidney)	Vomiting and diarrhea	Death	1

The absence (0) or presence (1) of the *adhB* gene was detected by PCR.

### *adhB* gene detection by PCR

For all the strains, a single colony was picked, resuspended in 100 μL Tris-EDTA NaCl buffer (TEN) and incubated at 98°C for 10 min. After centrifugation to pellet cell debris, 1 μl of supernatant was used as DNA matrix. The PCR mixture for gene detection contained 1 μl DNA matrix, 0.5 μM primer (forward: TTATTATCTATTCTTTCGTGTGATGC, and reverse CTATTTGTAGCAGAACATTC**R**AAACC), 10 μL DreamTaq Green PCR Master Mix (2X) (Thermo Scientific) in a final volume of 20 μL. Thermal cycling was carried out in a Mastercycler^®^ nexus (Eppendorf) with the following program: a start cycle of 3 min at 98°C, followed by 30 cycles of 20 s at 98°C, 30 s at 55°C, and 1 min at 72°C, and a final extension time of 10 min at 72°C. PCR fragment sizes were revealed on 1.5% agarose gels containing Midori Green, and visualised by a UV imaging device as previously described [26].

### *adhB* mutant generation

The Bt407 Cry^-^ with the reference genome *Bacillus thuringiensis Bt*407: NC_018877.1 was used as a model for *B*. *cereus* and was renamed Bc 407.

Knock-out of the *adhB* gene (WP_000438843) was accomplished by double-cross over gene substitution by use of the pMAD vector [32]. Briefly, using the available sequencing information of the Bc407 strain, 600 bp regions upstream and downstream of the identified gene of interest were synthesized surrounding a tetracycline-resistance cassette by the GeneCust company (Boynes, France). The upstream nucleotide coordinates used are 2,575,680 to 2,576,279, and the downstream nucleotide coordinates are 2,577,204 to 2,577,802. The synthesized region was then ligated into the pMAD vector. This vector was further transformed by heat shock into chemically competent NEB-10 beta cells. The plasmid was then extracted and transformed into *E*. *coli* strain ET to facilitate de-methylation of the plasmid, increasing subsequent transformation into *B*. *cereus Bc407* as previously described [16]. Resulting colonies were then subjected to temperature stress at 40°C to force the incorporation of the resistance cassette leading to the stable knock-out of the *adhB* gene, which was verified by PCR with oligonucleotide sequences flanking the cloned region. The mutation was stable and sequencing revealed that the mutation occurred at the corrected place and did not affect the flanking regions. The resulting strain was designated as Δ*adhB*.

Wild type and mutant strains were streaked onto BHI agar from 20% glycerol stocks to obtain isolated colonies. Colonies were inoculated into BHI broth and grown at 37°C, 200 rpm until mid to late-exponential phase for phenotypic analysis. Cultures in mid-exponential phase were used for microscopy to determine cellular morphology. For growth assays, stocks were inoculated into BHI broth and followed by sampling for CFU/ml at regular intervals.

### Nitric oxide (NO) stress survival

*B*. *cereus Bc407* and the Δ*adhB* mutant were grown to late-exponential phase. Cultures were harvested and diluted 1:1000 in RPMI (Gibco Glutamax, Fisher Scientific, Illkirch Cedex, France) and further grown at 37°C without agitation with differing doses of the NO donor, NOC-5 (3-[2-hydroxy-1-(1-methylethyl)-2-nitrosohydrazino]-1-propanamine (Calbiochem, Sigma-Aldrich, Saint-Louis, MO, USA). NOC-5 was dissolved in NaOH 0.01 M and used at the following concentrations: 0, 15.6, 25, 31.25, 50, 62.5, 100, 125, 250, 500 μM. After 1 h, bacteria were agitated to avoid sedimentation and the survival rate was quantified after 4 h by plating serial dilutions on LB agar plates. Data are pooled from two to four independent experiments and presented as % survival = (NO-treated/Buffer-treated) × 100.

### Oxidative stress survival

Oxidative stress-resistance was determined as previously described [33]. Briefly, wild-type and Δ*adhB* mutant strains were grown and 2 h post-inoculation, 500 μl of each culture was added to 100 μl of either sterile water or hydrogen peroxide at final concentrations of 2 mM or 10 mM. Treated (2 mM or 10 mM H_2_O_2_) and control (H_2_O) cultures were incubated for 10 min at 37°C and then serially diluted in phosphate-buffered saline (PBS) and plated on BHI to stop the reaction and count CFU/ml. Data are pooled from two independent experiments and presented as % survival = (H_2_O_2_-treated/H_2_O-treated) × 100.

### Insect infection trial

*B*. *cereus Bc407* and the Δ*adhB* mutant were grown to exponential phase. Cultures were harvested and serially diluted 1:4 in peptone water prior to injection. 10 to 20 last instar *Galleria mellonella* larvae were used following a 24 h fast as previously described [34, 35]. 10 μl of bacterial preparations at various doses were injected between the second and third body segment from the rear of the insect. Injected insects were incubated at 37°C for 24 h, following which survival was assessed. Peptone water was injected as negative control. Data are pooled from three independent experiments and presented as % survival = (injected with strain/injected with water) x 100.

### Protein bioinformatic analysis

The protein sequences of the ADH protein (WP_000438843) was analysed with Pfam to find functional domains. E-values are based on searching the Pfam-A family against UniProtKB 2018_04 using HMM search.

### Statistical analysis

Statistical analysis was performed with GraphPad Prism version 7. Insect survival curves were assessed by non-linear regression, constraining the bottom to 0.

Bacterial survival rate following stresses were also analysed by non-linear regression, and the statistical differences were calculated with a Wilcoxon test between the conditions with or without stress.

## Results

### *adhB* as a marker of clinical *B*. *cereus* strains

The presence/absence of the *adhB* gene was assessed by PCR on a collection of strains of varying pathogenic potential: 21 non-pathogenic strains and 35 clinical strains (Table 1). *adhB* was present in 34/35 (97%) clinical isolates, whereas it was present in 5 of 21 (24%) non-pathogenic isolates. We thus hypothesised that *adhB* may be a new and important virulence factor of *B*. *cereus*.

**Fig 1:**
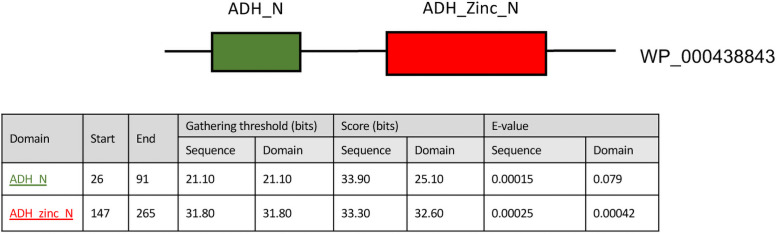
Structural domains of AdhB. The AdhB protein of *B*. *cereus* is composed of a catalytic domain with an inserted zinc-binding domain (green box) and a co-factor-binding domain at its C terminus (red box). E-values are based on searching the Pfam-A family against UniProtKB 2018_04 using hmmsearch.

The amino acid sequences of the Bc407 gene WP_000438843 coding for a protein of the AdhB family was analysed using the Uniprot database (Fig 1). This enzyme of 308 amino acids belongs to the zinc-containing alcohol dehydrogenase family. The software identified two domains, with the catalytic domain of the alcohol dehydrogenase containing an inserted zinc-binding domain. This domain has a GroES-like structure [36, 37]. The co-factor-binding domain of the enzyme is located proximal to the C-terminus. Structural studies indicate that it forms a classical motif called Rossman fold that reversibly binds NAD(H) as a co-factor [38, 39].

### Growth characteristics and morphology

**Fig 2:**
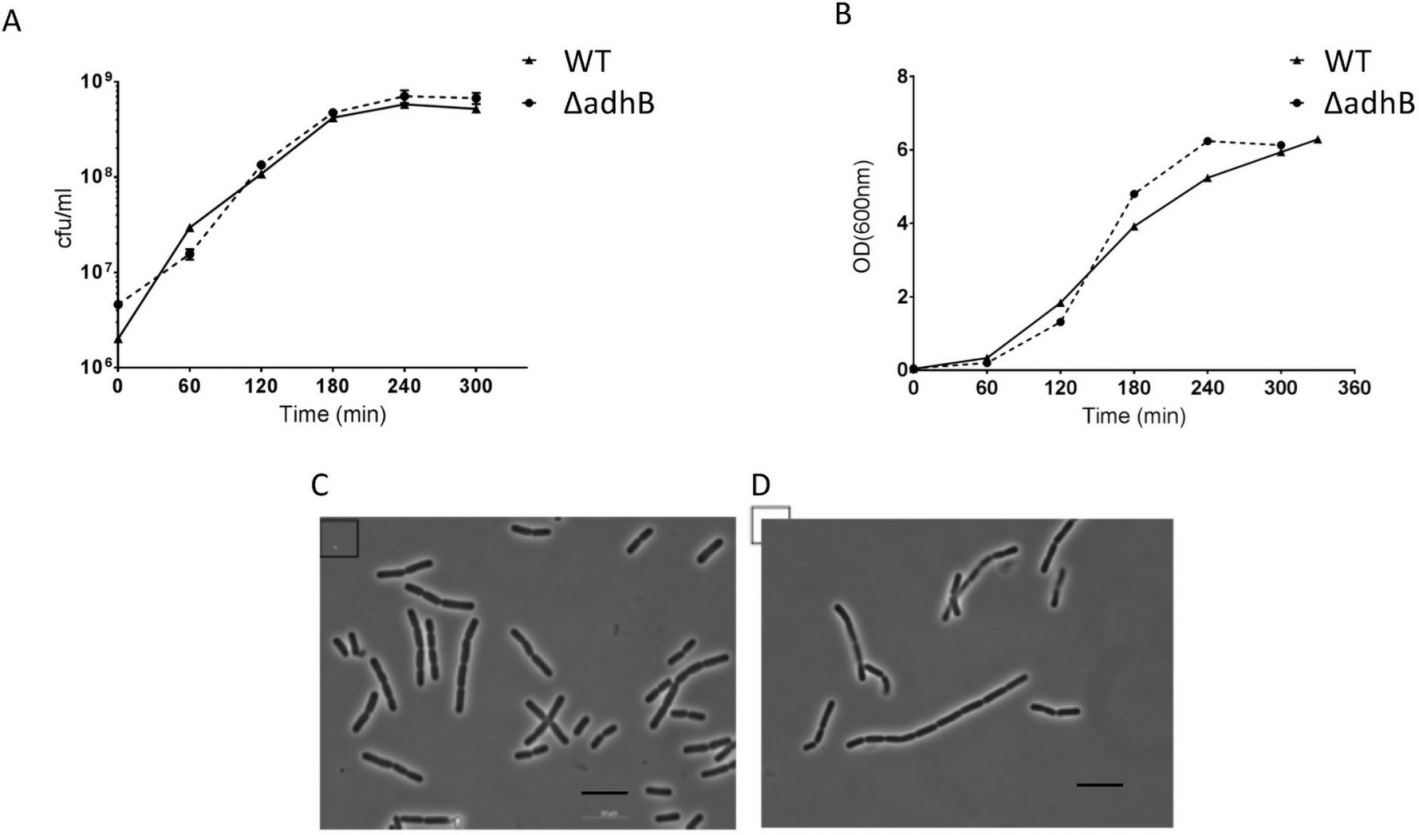
Bacterial growth curves and cellular morphology. Bacterial growth was determined by calculating CFU/mL (A) or following optical density at 600 nm (B) for the wildtype *B*. *cereus Bc407* (▲; solid line) and the Δ*adhB* mutant (●; dashed line) strains. Representative bacterial morphology of Bt407 WT (C) and *adhB* mutant (D) are viewed at 100x magnification. The scale bar represents a length of 10 μm. All graphs represent one representative experiment out of three biological replicates.

*B*. *cereus Bc407* and the Δ*adhB* mutant were grown in BHI medium at 37°C, 200 rpm and bacterial growth was followed by measuring the OD_600_, and CFU/mL determined by serial dilution and plating (Fig 2A and 2B). The two strains presented similar rates of growth with no significant differences in growth curves. The strains were observed under the microscope and bacterial morphology shows that the two strains are similar in cellular shape and size, with the *adhB* mutant often making longer chains of cells (6–8 cells) (Fig 2C and 2D).

### Nitric oxide (NO) and oxidative stress resistance

**Fig 3:**
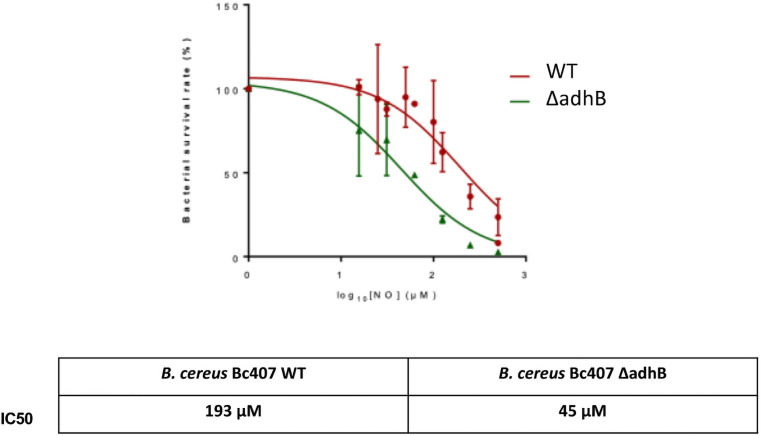
NO sensitivity. The wild type and Δ*adhB* mutant strains were cultured and incubated for 4 h in the presence of different concentrations of NO donors. Bacterial survival was quantified by plating and bacterial resistance to NO was measured and normalized with respect to the control condition, without NO. Data points correspond to the mean ± SEM of the values obtained from 2 to 4 biological replicates. The calculation of the IC50 of NO was performed using Graphpad.

To assess the role of AdhB in the resistance to the host immune system response, *B*. *cereus Bc407* and Δ*adhB* strains were incubated with the NO donor to test their resistance against NO stress (Fig 3). Several doses of NO were assessed and the dose inhibiting 50% of bacterial growth (IC50) was calculated. The IC50 of *B*. *cereus* wild type (WT) strain is approximately 4 times higher than that of the mutant (193 vs 45 μM of NO) and the survival rate of the mutant is lower at each concentration of NO tested. Thus, the mutant *adhB* is more sensitive to nitric oxide than the wild type strain.

**Fig 4:**
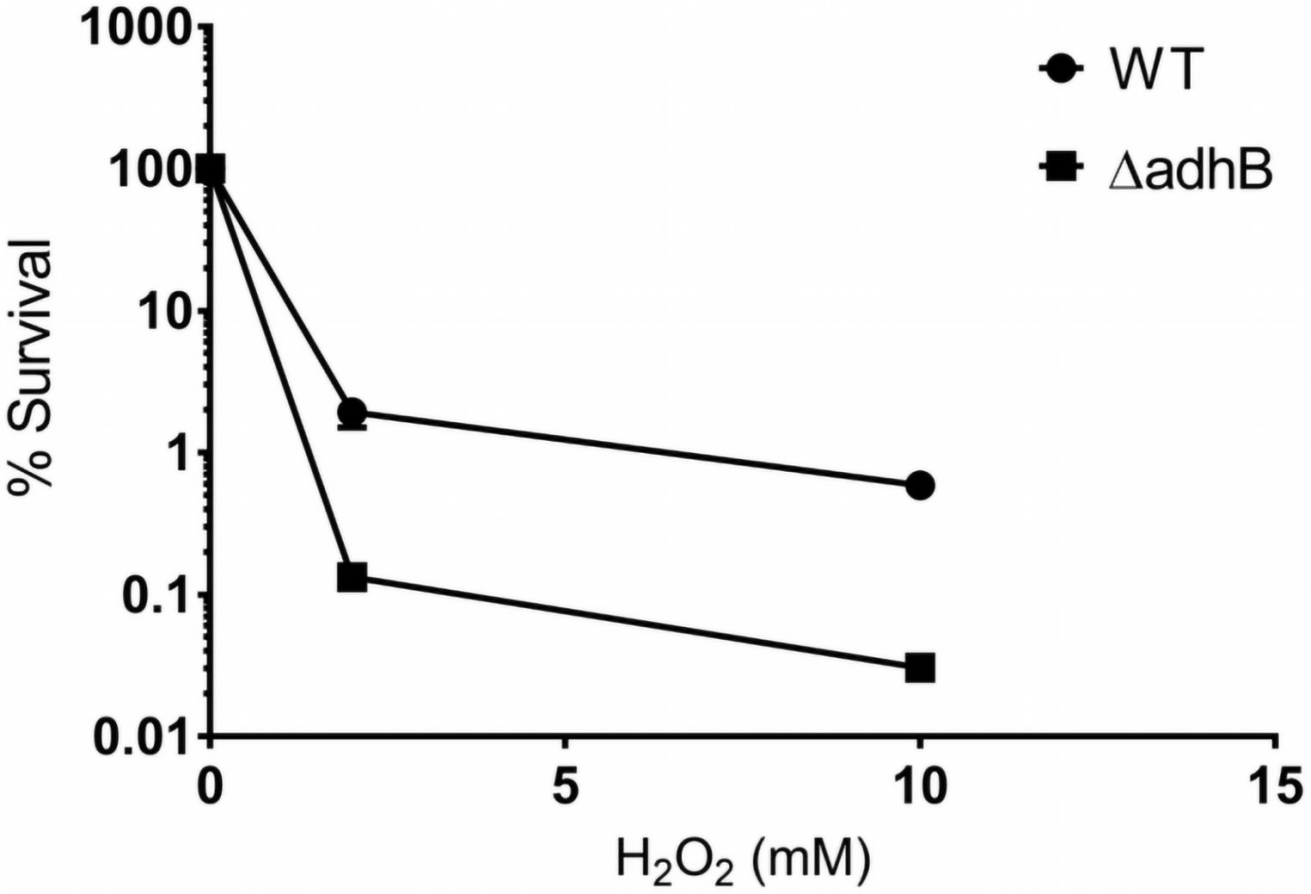
H_2_O_2_ sensitivity. The wild-type and Δ*adhB* mutant strains were grown and subsequently exposed to either 2 mM or 10 mM of hydrogen peroxide for 10 min at 37°C. Bacterial survival was assessed by plating and normalized against buffer-treated controls. Data points correspond to the mean ± SEM of the values obtained from 2 biological replicates.

Then, oxidative stress resistance of *B*. *cereus Bc407* WT and Δ*adhB* strains was determined after exposure to 2 mM or 10 mM H_2_O_2_ for 10 min at 37°C (Fig 4). Wildtype Bc407 demonstrated increased resistance at both concentrations, with survival percentage being 14-fold higher at 2 mM, and 20-fold higher at 10 mM.

### Insect model of *B*. *cereus* toxicity

**Fig 5:**
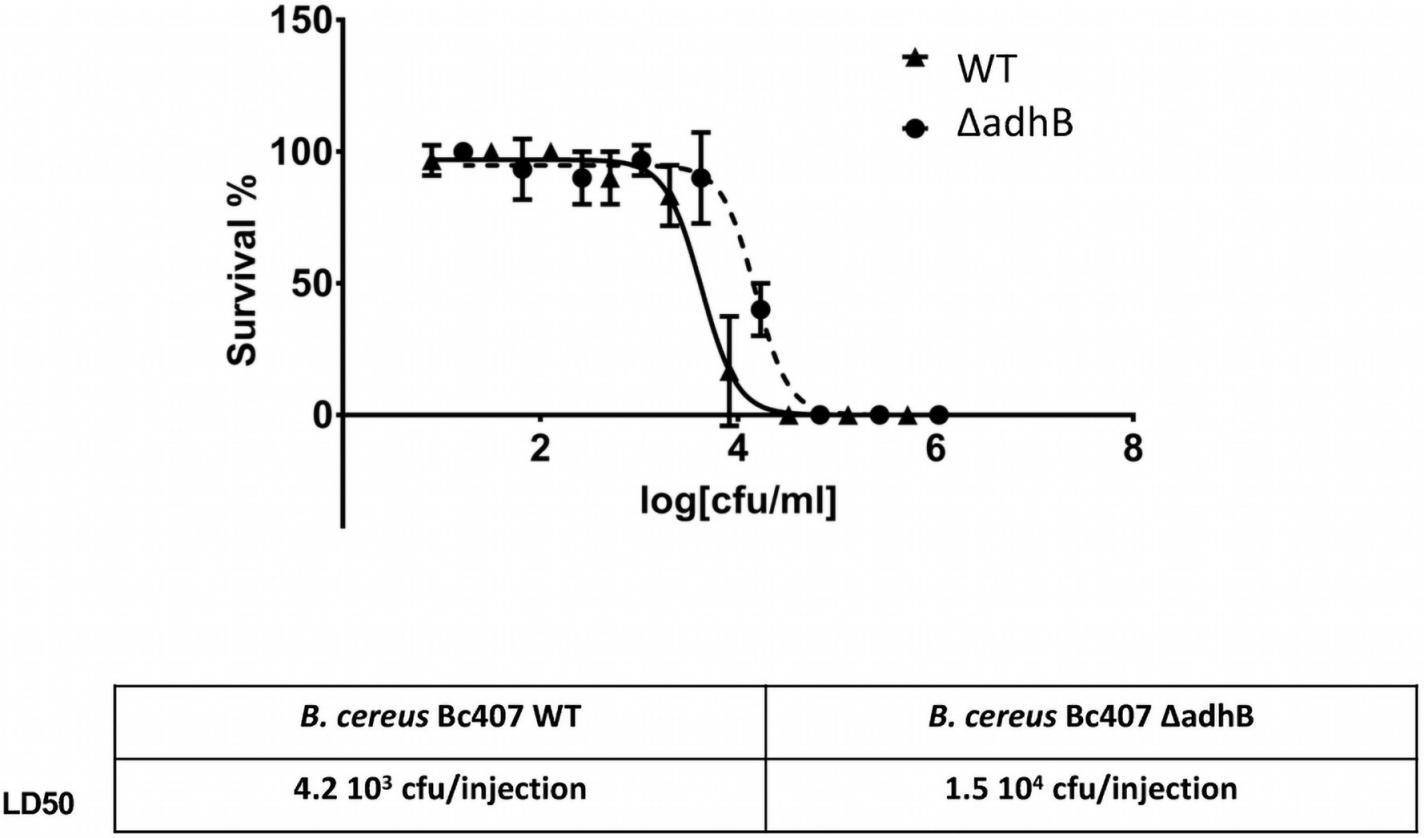
Insect infection. Bacterial virulence was determined as *Galleria mellonella* survival percentage following injection with varying CFU/mL of wild type (triangles, black line) or Δ*adhB* (circles, dashed line) mutant strains. Survival was measured as live insects following 24 h post-injection. Calculation of the LD50 was done using Graphpad software.

The role of AdhB in the pathogenicity of *B*. *cereus* was assessed in an insect model of infection. *B*. *cereus Bc407* and Δ*adhB* mutant strains were injected at various doses into *Galleria mellonella* larvae (Fig 5). At 24 h post-injection, survival of the insects was assessed. Insects infected with the Δ*adhB* mutant strain demonstrated higher rates of survival in relation to the wildtype strain, demonstrating a reduced virulence of the mutant strain. Further, statistical analysis of the survival curves reveals a significant difference in the LD50 values between the strains: 4.2 10^3^ CFU/injection for the wildtype and 1.5 10^4^ CFU/injection for the Δ*adhB* mutant. HillSlope determined the curves to be distinct at 99.94% probability.

## Discussion

**Fig 6:**
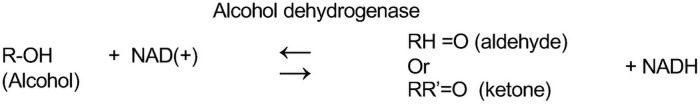
Reaction catalyzed by an alcohol dehydrogenase. The alcohol dehydrogenase catalyzes the oxidation of alcohol into their corresponding aldehyde (primary alcohol) or ketone (secondary alcohol) with the reduction of NAD+.

Alcohol dehydrogenase (ADH) is an enzyme involved in oxidation-reduction biological process. It catalyses the reversible oxidation of alcohols and induces the formation of their corresponding acetaldehyde or ketone with the reduction of NAD (Fig 6). This class of enzyme typically has a broad spectrum of action [40, 41]. Here we characterized AdhB as a protein involved in *B*. *cereus* resistance to nitric and oxidative stresses, two major components of the host immune system, and in its pathogenicity.

Currently three types of alcohol dehydrogenases are known, that differ structurally and catalytically: Zinc-containing ’long-chain’ alcohol dehydrogenases, ’short-chain’ alcohol dehydrogenases, and iron-containing alcohol dehydrogenases [42, 43]. The AdhB (WP_000438843) protein in *B*. *cereus* is a zinc-containing ADH. These enzymes are typically dimeric or tetrameric proteins, which require two atoms of zinc per subunit to be functional, however, catalytic activity is maintained in the presence of a single zinc atom. The zinc atoms interact with either cysteine or histidine residues; the catalytic zinc being coordinated by two cysteines and one histidine. Zinc-containing ADH’s are found in bacteria, mammals, plants, and fungi. Normally, there is more than one isozyme per species (e.g. humans possess at least six isozymes and yeast have three). Consistently, we identified three Zinc-containing ADH’s in the Bc407 strain (WP_000438843, WP_000649129.1, WP_000645827.1). These three isozymes share common structures with two identified domains (not shown). The first is the catalytic domain that might contain an inserted zinc-binding domain. This domain has a GroES-like structure; a name derived from the superfamily of proteins with a GroES fold. Proteins with a GroES fold structure have a highly conserved hydrophobic core and a glycyl-aspartate dipeptide, which is thought to maintain the fold. The second is the domain that binds its cofactor NAD owing to its motif denoted as a Rossman fold [38, 39].

In order to specify the role of AdhB in *B*. *cereus*, the virulence of the wild type and Δ*adhB* mutant was tested in an insect infection model. *G*. *mellonella* larvae were used as a model of infection as *B*. *cereus* is both a human and an insect pathogen [25, 44]. This study reveals that *adhB* plays an essential role during *B*. *cereus* virulence and could thus be considered as a new pathogenic factor.

During human or insect infections, *B*. *cereus* is able to resist the host immune system and persist. It can indeed survive phagocytosis by macrophages and can induce their apoptosis [20, 45]. The primary mechanism of macrophage-induced cytotoxicity is through the massive production of nitric oxide and oxidative stress at the peak of inflammation leading to bacterial death [46, 47]. Thus, bacterial response to NO is of major importance for bacterial survival and several pathogenic bacteria have developed means for detoxification and repair of the damages caused by NO [48]. We have previously shown that *B*. *cereus* is particularly resistant to NO [15, 18, 45, 49]. Here, we show that the Δ*adhB* mutant was more sensitive than the wildtype strain to both oxidative and nitric stresses. Accordingly, this sensitivity may be implicated in the reduced mutant virulence in the insect model.

The initial step of bacterial response to NO and oxidative response is the detection of reactive oxygen and nitrogen species (ROS and RNS), which will permit to activate the detoxification and repair pathways. It has been previously shown that virulence factor production by *B*. *cereus* is dynamic and shaped by cellular oxidation [50]. ADH proteins have been previously shown to be involved in the reduction of alcohol and the production of NADH. NADPH is required to maintain and regenerate the cellular detoxifying and anti-oxidative defense systems [51]. The antioxidant defense system of *B*. *cereus* is constituted by an elaborate, often overlapping network of enzymes [52], but to the best of our knowledge, there was no evidence of ADH implication in the resistance of oxidative or NO stress. As oxidative and NO response overlap during the immune response, it is not surprising that mechanisms of bacterial resistance against ROS and RNS share similarities. The reduction capacity of ADH may be involved in NO detoxification. Bacterial capacity to detoxify NO through reduction is widely distributed in denitrifying bacteria but is also present in pathogens. For denitrifying bacteria, the reduction of nitrate to N_2_ is part of the nitrogen cycle and prevents NO high toxicity; for pathogenic bacteria, NO detoxification might be a mean to survive under oxygen limited environments and to survive to nitrogen stress [46, 47, 53].

Taken together, we have identified a new virulence factor implicated in *B*. *cereus* resistance to host immunity whose activities may explain the pathogenic potential of clinical strains carrying this newly identified pathogenic biomarker.
